# Screening of Phospholipids in Plasma of Large-Artery Atherosclerotic and Cardioembolic Stroke Patients With Hydrophilic Interaction Chromatography-Mass Spectrometry

**DOI:** 10.3389/fmolb.2022.794057

**Published:** 2022-01-20

**Authors:** Haixing Wang, Siyan Chen, Zhao Han, Ting Li, Jianfeng Ma, Xi Chen, Jie Pang, Qingcheng Wang, Qing Shen, Manman Zhang

**Affiliations:** ^1^ Zhejiang Provincial Key Laboratory of Anesthesiology, Department of Anesthesiology, The Second Affiliated Hospital of Wenzhou Medical University, Wenzhou, China; ^2^ Department of Neurology, The First Affiliated Hospital of Wenzhou Medical University, Wenzhou, China; ^3^ Department of Neurology, The Second Affiliated Hospital of Wenzhou Medical University, Wenzhou, China; ^4^ Clinical Research Unit, The Second Affiliated Hospital of Wenzhou Medical University, Wenzhou, China; ^5^ Department of Cardiology, Zhejiang Provincial People’s Hospital, People’s Hospital of Hangzhou Medical College, Hangzhou, China; ^6^ Hangzhou Linping Hospital of Traditional Chinese Medicine, Hangzhou, China; ^7^ Collaborative Innovation Center of Seafood Deep Processing, Zhejiang Province Joint Key Laboratory of Aquatic Products Processing, Institute of Seafood, Zhejiang Gongshang University, Hangzhou, China

**Keywords:** ischemic stroke, lipidomics, diagnosis, mass spectrometry, phospholipid

## Abstract

Ischemic stroke (IS) is a deadly and debilitating disease with a high incidence and recurrence rate in elderly people worldwide. Large-artery atherosclerotic (LAA) and cardioembolic (CE) stroke are two leading subtypes and require different management. As a complementary biochemistry method for current diagnostic techniques, a sensitive and accurate phospholipid (PL) targeted lipidomic method was developed in this study. Plasma PLs were selectively extracted with titanium dioxide/fibrous silica nanosphere material, then characterized and quantified with hydrophilic interaction chromatography-mass spectrometry. A total of 31 molecular species of PLs were determined and ten biomarkers including seven molecular species of sphingomyelins (SM d18:1/18:1, d18:1/18:0, d18:1/24:1, d18:1/16:1, d18:1/22:1, d18:1/24:2, and d18:1/16:0) and three molecular species of phosphatidylcholines (16:0/18:1, 16:0/18:2 and 16:0/22:6) showed significant differences in LAA, CE, and healthy control (HC) groups. The independent diagnostic capabilities of these PL biomarkers were successfully evaluated and validated with receiver operating characteristic curves. Additionally, the oleic acid-enriched SMs, which can result in atherogenic lipoprotein aggregation, were proved to be positively related to IS and may perform as the potential risk factors in the future. Meanwhile, valuable suggestions for dietary interventions as an essential source of endogenous PLs could be obtained from this study.

**Graphical Abstract:**
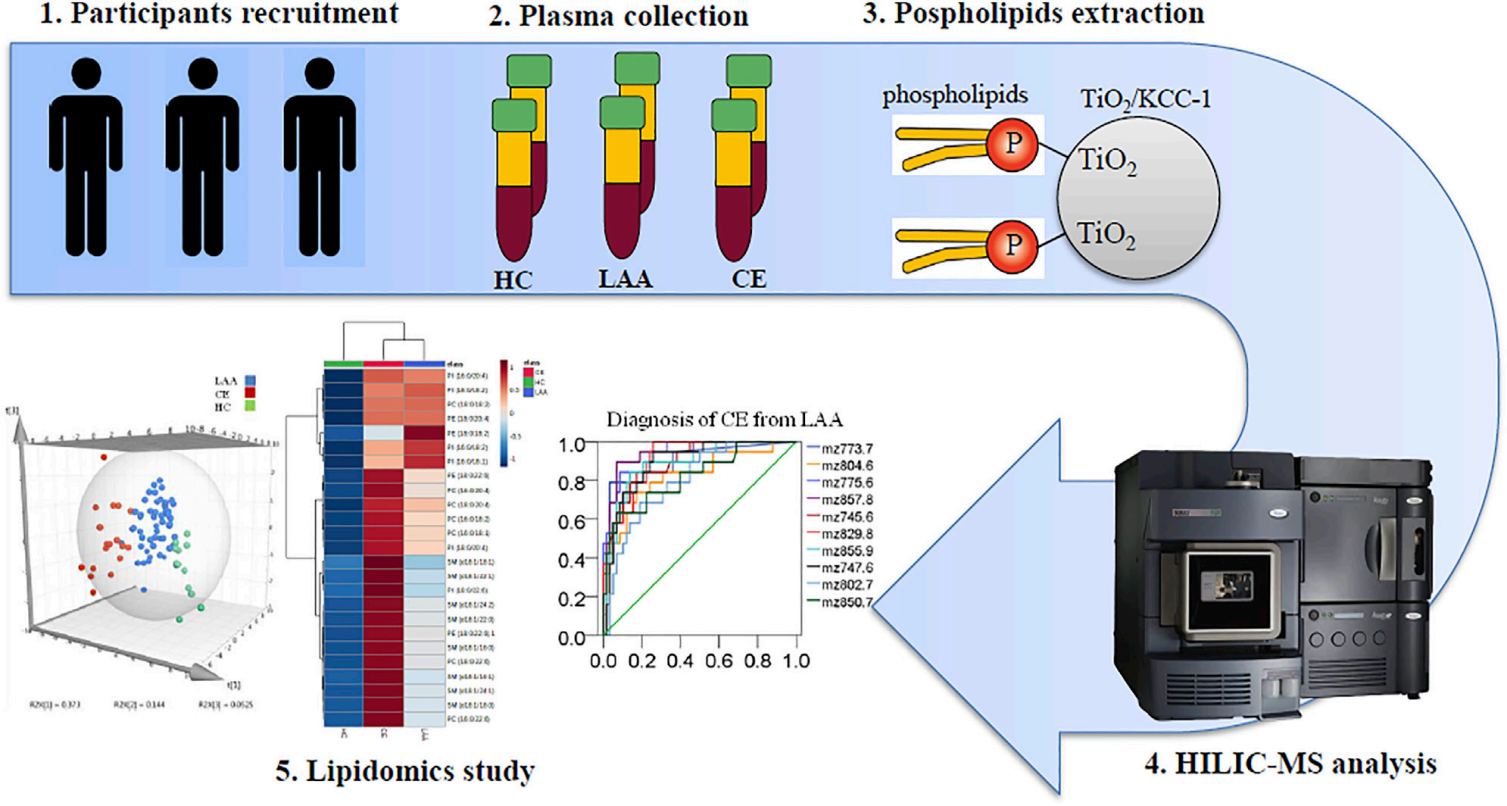


## Introduction

Ischemic stroke (IS) is a neurologic emergency resulting from an abrupt decrease and blockage of blood supply. IS can cause brain tissue damage and focal neurological function impairments. According to WTO reports, stroke is now one of the world’s leading contributors to death and disability. The reoccurrence rate of IS is high and regimens of secondary prevention depend on the reliable identification of the etiology. The Trial of Org 10172 in Acute Stroke Treatment (TOAST) classification categorizes IS into five subtypes according to etiology: large-artery atherosclerosis (LAA), cardioembolic (CE), small vessel occlusion, undetermined, and other specific etiologies (Adams et al., 1993). As the two most important and conventional subtypes accounting for half of acute IS cases, LAA and CE have received great attention and the related pathophysiologies have been well-documented, which are also the basis of the choice of current secondary prevention strategies in clinical practice. According to current guidelines, antiplatelet agents are recommended for the LAA subtype resulting from atherosclerosis and endothelial injury, whereas anticoagulant therapy is preferred for the CE subtype, primarily attributed to cardiogenic embolism (Del Brutto et al., 2019). Thus, the IS subtype diagnosis is crucial for drawing up proper management to prevent patients from a second or even a third attack. Current strategies for analyzing the specific etiology of an ischemic event with imaging technologies and clinical evaluation are experience-dependent and insufficient at some point. The blood test for IS diagnosis is not available yet (Goulart et al., 2019). Therefore, a new complimentary assessment approach, metabolomic-dependent plasma analysis, is developed to make etiology diagnosis of acute IS more accurately and efficiently.

Phospholipids (PLs) are a class of amphiphilic lipids whose molecule has one polar phosphate “head”, and two no-polar fatty acid tails, which are joined by a glycerol molecule. Besides acting as the main component of the cell membrane, PLs play essential roles in many cellular processes including cell metabolism and respiration, lipid absorption, blood coagulation, and various biological processes (Castagna et al., 1982; Harayama and Riezman, 2018). Currently, PLs have been continually proved to be associated with some complex diseases, including cardiovascular disease and cancer (Jiang et al., 2000; Aglago et al., 2021). So the availability of PLs originating from both dietary intake and enzymes controlled endogenous metabolism, is of significance to health (Küllenberg et al., 2012; Simopoulos, 2013). Recent development in metabolomics technologies provides new insight into the identification of plasma biomarkers across different diseases. Increasing evidence shows that plasma PL molecular species, including phosphatidylcholine (PC), phosphatidylethanolamine (PE), phosphatidylinositol (PI), phosphatidylserine (PS), and sphingomyelin (SM), played essential roles in the development of IS (Ke et al., 2019; Sidorov et al., 2019). For example, clinical researches revealed alterations of various metabolic PL biomarkers, such as PC, PE, lysoPC, and lysoPE, in IS patients compared with healthy control (HC) subjects (Ke et al., 2019); A metabolic product of PLs, lysophosphatidic acid, had been frequently documented to participate in multiple processes and involved in platelet aggregation, atherogenesis, and thrombogenic activity (Korporaal et al., 2001; Chung et al., 2007; Li et al., 2020). Adibhatla et al. found that CDP-choline could significantly restore PC level by differentially affecting secretor phospholipase A (2) (sPLA(2)-IIA), ptdCho-specific phospholipase C (PtdCho-PLC), and phosphocholine cytidylyltransferase (CCT) alpha after transient focal cerebral ischemia (Adibhatla et al., 2006). At the same time, Simon et al. considered that the level of fatty acid alpha-linolenic acid in PLs was independently associated with the risk of stroke (Simon et al., 1995). Kim et al. and Chun et al. found that the composition of fatty acids in plasma PLs was associated with intracranial atherosclerotic stenosis-reduced-IS and cardioembolic stroke in the Korean population (Kim et al., 2012; Chung et al., 2015). It is well known that the ω-3 and ω-6 polyunsaturated fatty acids together with their oxidated metabolites are correlated with the happening and resolution of inflammation as pro-inflammatory and pro-resolving lipid mediators (Serhan, 2014; Zhuo et al., 2018). Admittedly, the inflammatory response is a major factor in stroke pathobiology and outcome (Anrather and Iadecola, 2016). PLs are the essential resource of circulating fatty acids and the decline of the physiological reserve is vital in many age-related diseases including IS. Therefore, we sought to explore the etiology of IS with PL molecular species and seek the PL biomarker for differentiating and diagnosing IS subtypes.

Mass spectrometry (MS) is an analytical technique according to the mass-to-charge ratios (*m/z*) of ions, which is usually coupled with high-performance liquid chromatography (HPLC) for the analysis of complex samples after chromatographic separation. Due to its high sensitivity and specificity, HPLC-MS has been widely applied in clinical laboratories for drug metabolism and pharmacokinetics (DMPK), metabolomics, proteomics, and disease diagnosis. Hydrophilic interaction chromatography (HILIC) is a kind of HPLC technique that has been reported for the separation of PLs in lipidomics (Zhao et al., 2011; Zhu et al., 2012). The hydrophilic stationary phase in the HILIC column can interact with the polar head regions of the PL molecules, and different classes of PLs are separated and eluted from the column in sequence. The organic mobile phase used for elution in HILIC leads to high signal resolution and sensitivity. In our previous studies, HILIC-MS had been successfully applied in the PL-based lipidomic analysis of seafood (Shen et al., 2021a; Shen et al., 2021b). Because of the chelating bidentate bonds between the phosphate group and metal oxide, titanium dioxide (TiO_2_) could be applied to extract PLs and phosphopeptides from biological samples (Ikeguchi and Nakamura, 2000; Engholm-Keller and Larsen, 2011). Recently, a more effective and efficient chromatographic material TiO_2_/fibrous silica nanosphere (KCC-1), was synthesized in our laboratory and successfully applied to extract PLs from tissue samples (Zhang et al., 2021).

In this study, solid-phase extraction (SPE) using laboratory-made TiO_2_/KCC-1 was employed to extract PLs from human plasma. Then a HILIC-MS method was conducted to characterize and quantify different molecular species of plasma PL. With the assistance of lipidomics and statistics, we planned to identify the exact PL molecular species related to LAA and CE stroke, which could be applied as potential biomarkers for subtype differentiation or diagnosis and provide helpful suggestions for dietary intervention to reduce the risk of stroke to some extent.

## Materials and Methods

### Reagents and Materials

HPLC grade isopropanol, methanol (MeOH), and acetonitrile (ACN) were purchased from Merck Millipore (United States). Formic acid was purchased from Sigma-Aldrich (United States). PL standards l,2-dipalmitoyl-sn-glycero-3-phosphocholine (PC 16:0/16:0), l,2-distearoyl-sn-glycero-3-phosphoethanolamine (PE 18:0/18:0), N-lignoceroyl-D-erythro-sphingosylphosphorylcholine (SM d18:1/24:0) and l-stearoyl-2-arachidonoyl-sn-glycero-3-phosphoinositol (PI 18:0/20:4) (ammonium salt) were purchased from Avanti Polar Lipids (Alabama, United States). Ultrapure water (18.0 MΩ cm at 25°C) was obtained from a Milli-Q water system (Millipore, United States).

### Plasma Sample Collection

Ethical approval was gained from the Research Ethics Committee of The First Affiliated Hospital of Wenzhou Medical University (reference number: 2018-131). Informed consent about the use of the blood samples for scientific research purposes was obtained from the recruited patients (>18 years of age).

Participants were recruited among patients admitted into The First Affiliated Hospital of Wenzhou Medical University between March 2020 and September 2020. The blood sample was collected within 3 days of the onset of acute IS which was diagnosed based on the clinical presentation and a relevant lesion on brain magnetic resonance imaging. Age-matched healthy volunteers free of vascular disease were enrolled as the HC group. IS participants were further classified as LAA and CE groups using the TOAST classification in this study. Baseline characteristics of the study groups were presented in Table 1.

**TABLE 1:** Baseline characteristics of the studied subjects.

	Control (N = 12)	Ischemic stroke	
		CE (N = 19)	LAA (N = 58)
Sex (Male)	7 (58.30%)	12 (63.20%)	37 (63.80%)
Age	61.9 ± 14.2	71.1 ± 12.9	67.4 ± 12.5
BMI, kg/m^2^	22.8 ± 2.9	24.3 ± 2.9	23.7 ± 3.8
Alcoholism	2 (16.67%)	2 (10.50%)	17 (29.30%)
Smoking	4 (33.33%)	6 (31.60%)	25 (43.10%)
Coronary artery disease	0 (0.00%)	3 (15.80%)	1 (1.70%)
Diabetic mellitus	3 (25.00%)	4 (21.10%)	16 (27.60%)
Previous stroke	2 (16.70%)	4 (21.10%)	14 (24.10%)
Systolic BP, mm Hg	142.3 ± 18	142.8 ± 16.3	151.1 ± 18.7
Diastolic BP, mm Hg	79.9 ± 11.5	83.5 ± 10.4	85.7 ± 13.4
eGFR, mL·min^−1^ 1.73m^−2^	83.7 ± 35.1	78.7 ± 23.5	89.7 ± 20.6
Fibrinogen, g/L	3.2 ± 0.7	3.2 ± 1	3.8 ± 1.2
HbA1c, %	6.9 ± 1.5	6.4 ± 1.4	7.1 ± 1.9
Homocysteine, μmol/L	14.0 ± 3.6	17.8 ± 7.8	15.1 ± 10.1
mRS	—	3.1 ± 1.5	2.5 ± 1.5
NIHSS	—	5.7 ± 6.4	4.4 ± 4.1

All data were expressed as number or mean ± SD.

### Sample Preparation

A modified Folch method was employed to extract crude lipids from human plasma firstly. A portion of 100 μL of plasma was added with 1.5 ml of Folch solution chloroform/methanol (2:1, v/v). The mixture was shaken in a vortex for 2 min, sonicated for 15 min, and centrifuged at 12,000 g in 8°C (Eppendorf 5424R, Germany) for 5 min. When the insoluble portion was removed, 0.2 ml of water was added into the mixture to induce phase separation after another cycle of vortex mixing and centrifugation. The lower organic phase was carefully separated and freeze-dried by evaporation under vacuum to get the residue of crude lipids, which was stored at −80°C before further preparation.

The PL components were further extracted with a laboratory-made TiO_2_/KCC-1 SPE column (10 mg/1 ml cartridge) referring to a previously reported SPE method from our group (Zhang et al., 2021). Briefly, the SPE column was activated by methanol and 0.1% FA in water in the beginning. The total lipids residue was transferred to the SPE column with the necessary amount of Folch solution. After sample loading, the SPE column was washed with chloroform/2-propanol (2:1, v/v) and 10% MeOH aqueous solution to remove fatty acids and neutral lipids without phosphate group. The targeted PL fraction was eluted from the SPE column with chloroform/methanol (1:2, v/v) and dried under a vacuum. The obtained PLs residue was redissolved in 500 μL of Folch solution and filtrated with the 0.22 μm polytetrafluoroethylene membrane before HPLC-MS analysis.

### HPLC-MS Analysis

An HPLC system (Waters, ACQUITY H-Class, Milford, MA, United States) was employed for chromatographic separation with a Cosmosil HILIC column (5 μm, 150 mm × 4.6 mm, Nacalai Tesque, Japan) at 30°C. Ultrapure water containing 20 mM ammonium formate and 0.1% formic acid was used as mobile phase A, and ACN was used as mobile phase B. The flow rate of gradient elution was 0.6 ml min^−1^ and the final optimized conditions were given as follows: 0.0 min, 5% A - 95% B; 3.0 min, 5% A - 95% B; 4.0 min 12% A - 88% B; 10.0 min 12% A - 88% B; 12.0 min 50% A - 50% B; 17.0 min 50% A - 50% B. The sample bottles were temporarily stored in the autosampler at 8°C, and 1 μL of sample solution was injected into the HPLC system for analysis.

A triple-quadrupole mass spectrometer (Waters, Xevo TQD, Manchester, United Kingdom) was coupled with the HPLC system for mass spectrometric analysis. The MS equipment was operated using an electrospray ionization (ESI) method in negative ionization mode. The MS scan function was applied for formal analysis with a scan time at 1 s and the scanning mass range at *m/z* 600-1,000. The daughter ions scan function was applied for tandem mass spectrometric (MS/MS) analysis with optimized collision energy at 22–40 V, and the scan time was set as 0.1 s. Argon was employed as the collision gas. The other general MS parameters were given as follows: capillary voltage, 4.0 kV; cone voltage, 30 V; desolvation temperature, 500°C; desolvation gas flow rate, 1000 L h^−1^; cone gas flow rate, 30 L h^−1^.

### Data Acquisition and Statistical Analysis

The HPLC-MS data were acquired with the MassLynx V4.1 software (Waters, United Kingdom). Statistical analysis was performed with Microsoft Office Excel (version 2007, United States), SIMCA (version 14.1, MKS Umetrics, Sweden), and SPSS Statistics (version 19, IBM Company, United States).

In order to analyze the PL differences among LAA, CE, and HC groups, a one-way analysis of variance (ANOVA) method was applied with a significance level of 0.05 (95% CI). Levene’s test was used to evaluate the equality of variances. If the variances between groups were equal (homogeneity of variance), the least-significant difference (LSD) was calculated to examine the statistical significance. If the variances between the three groups were not equal, the Tamhane’s T^2^ test was performed. The *p*-value ≤ 0.05 means the differences are statistically significant.

## Results and Discussion

### Study of Participant Characteristics

In this study, a total of 89 subjects (12 HC, seven male, age 61.9 ± 14.2; 19 CE patients, 12 male, age 71.1 ± 12.9; 58 LAA patients, 37 male, age 67.4 ± 12.5) were included. As shown in Table 1, there were no statistical differences found in sex, age, body mass index (BMI), alcoholism, smoking, history of diabetic mellitus, previous stroke, coronary artery disease, systolic blood pressure (BP), diastolic BP, estimated glomerular filtration rate (eGFR), fibrinogen, Hemoglobin A1C (HbA1c), homocysteine, modified Rankin Scale (mRS) and National Institute of Health Stroke Scale (NIHSS).

### Characterization and Quantification of PLs in Human Plasma

In order to perform PL profiling with HPLC-MS, plasma PL metabolites were extracted and purified firstly. Conventional lipid extraction methods are Folch, Bligh & Dyer, and methyl-tert-butylether (MTBE) (Ranjith Kumar et al., 2015). The Folch method had been proved to suit the extraction of a broad range of lipid classes from biological samples (Ranjith Kumar et al., 2015; Ulmer et al., 2018). Therefore the Folch method was chosen and optimized. Different from cholesterol esters and triglycerides, PLs have an exclusive phosphate group that can bind with TiO_2_. Since the mesoporous KCC-1 nanoparticle has a wrinkly structure that significantly increases its surface area, TiO_2_ was coated on the surface to improve PL extraction efficiency. The TiO_2_/KCC-1 material was synthesized using a previously reported method by our group and the highest recoveries of PC, PE, and PI extraction were obtained in the range of 60.2–80.1% (Zhang et al., 2021). Additionally, the SPE parameters including pH, eluent volume, and flow rate were investigated and decided in our previous study. For this reason, the obtained crude lipids residue was further treated with an SPE method using laboratory-synthesized chromatographical material TiO_2_/KCC-1 to extract and purify the plasma PL metabolites.

**FIGURE 1:**
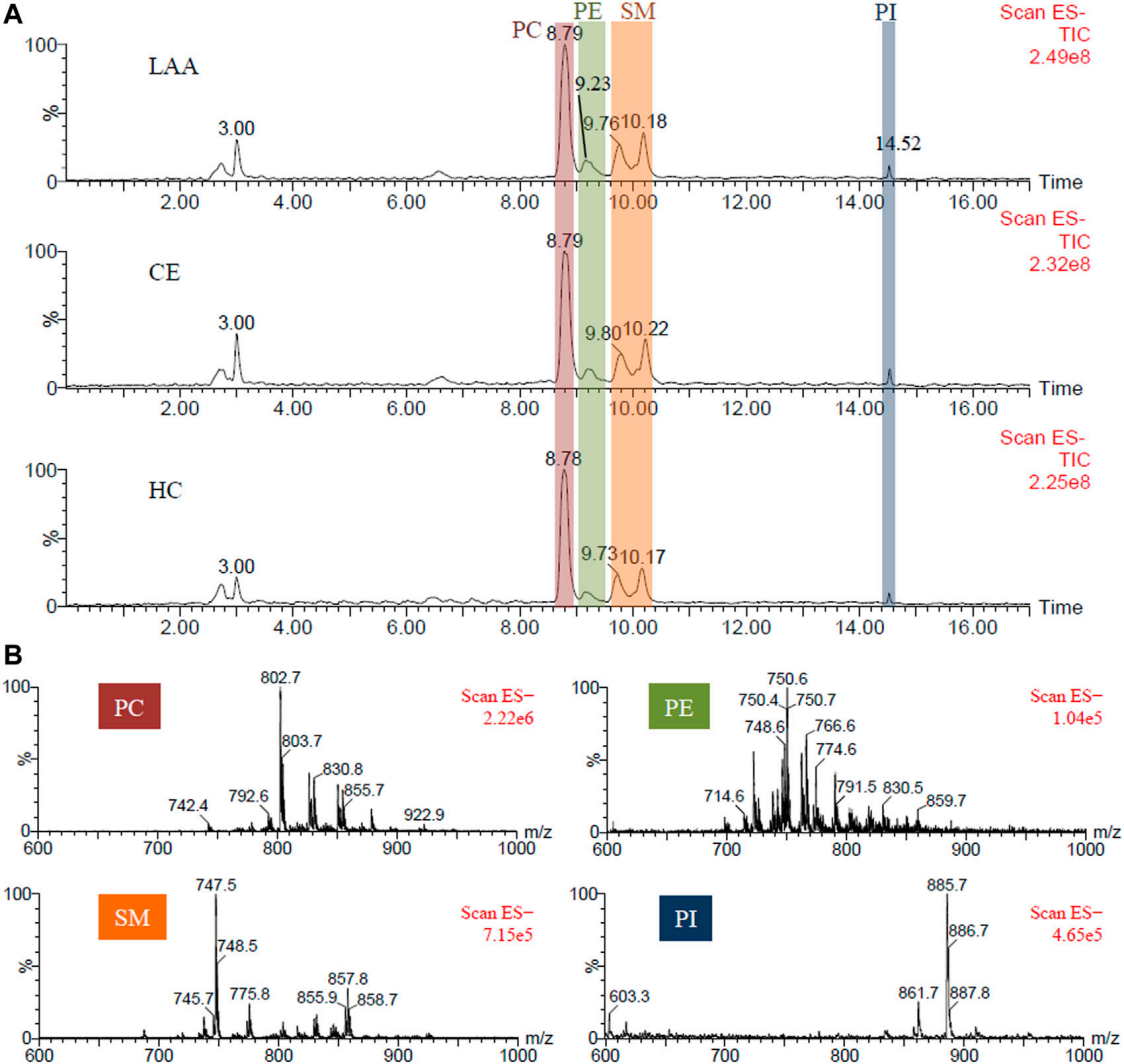
**(A).** The representative TIC chromatograms of PLs extracted from the plasma of LAA and CE patients, and HC participants respectively; **(B)** The mass spectra of PC, PE, SM, and PI components.

In order to facilitate the characterization of PL molecule species, a triazole HILIC column was employed for normal-phase chromatographic separation according to the polarity discrepancy of their head groups (Shen et al., 2020). PLs could be eluted from a HILIC column in order of class (Zhu et al., 2012). Once the PL class was determined, the length and unsaturation degree of the fatty acid chains could be easily determined according to the molecule weight. Referring to the retention time of PL standards, PC, PE, SM, and PI in human plasma were eluted from the HILIC column as shown in the total ion chromatogram (TIC) in Figure 1A. Corresponding mass spectra of different PL classes were given in Figure 1B. In Table 2, the exact molecules of PLs were further characterized according to their fatty acid chains’ fragment ions in tandem mass spectrometry (MS/MS) spectra. For example, the predominant ion *m/z* 802.7 of a PC molecular species at 8.79 min produced two fragment ions at *m/z* 255.2 (palmitic acid, FA 16:0) and *m/z* 279.7 (linoleic acid, FA 18:2) in MS/MS spectrum (Supplementary Figure S1). So *m/z* 802.7 was identified as the formate adduct ion [M + COOH]^−^ of PC (16:0/18:2). Corresponding ions at *m/z* 742.8 and *m/z* 792.6 were identified as its demethyl ion [M-CH_3_]^−^ and chloride adduct ion [M + Cl]^−^ respectively according to their MS/MS spectra and the *m/z* values.

**TABLE 2:** The characterized molecular species of PLs and their concentrations in human plasma.

	*m/z*	Identified PLs	Concentrations (mean ± SD, μg/mL)		
			LAA	CE	HC
PC	742.8 [M−CH_3_]^−^	PC (16:0/18:2)	82.4 ± 14.0	97.5 ± 13.6	59.5 ± 11.6
	792.6 [M+Cl]^−^				
	802.7 [M+COOH]^−^				
	804.6 [M+COOH]^−^	PC (16:0/18:1)	44.7 ± 7.4	54.2 ± 7.7	29.3 ± 6.3
	816.7 [M+Cl]^−^	PC (16:0/20:4)	30.9 ± 9.3	36.7 ± 9.5	24.4 ± 7.2
	826.7 [M+COOH]^−^				
	820.8 [M+Cl]^−^	PC (18:0/18:2)	36.1 ± 7.2	35.9 ± 7.9	26.6 ± 6.1
	830.9 [M+COOH]^−^				
	840.7 [M+Cl]^−^	PC (16:0/22:6)	14.5 ± 4.9	22.4 ± 6.7	8.7 ± 3.2
	850.7 [M+COOH]^−^				
	844.7 [M+Cl]^−^	PC (18:0/20:4)	23.7 ± 7.2	25.9 ± 7.1	18.7 ± 6.5
	854.7 [M+COOH]^−^				
	868.8 [M+Cl]^−^	PC (18:0/22:6)	5.2 ± 3.0	8.7 ± 3.8	2.2 ± 1.6
878.7 [M+COOH]^−^					
PE	714.6 [M−H]^−^	PE (16:0/18:2)	0.6 ± 1.6	0.0 ± 0.2	0.0 ± 0.2
	722.5 [M−H]^−^	PE (p-14:0/20:4)	2.6 ± 3.4	1.2 ± 1.9	2.0 ± 2.9
	738.5 [M−H]^−^	PE (16:0/20:4)	0.6 ± 1.4	0.5 ± 0.8	0.1 ± 0.2
	742.6 [M−H]^−^	PE (18:0/18:2)	4.4 ± 5.1	2.2 ± 2.7	0.5 ± 1.1
	748.6 [M−H]^−^	PE (p-16:1/20:4)	3.6 ± 3.8	2.8 ± 2.7	2.4 ± 3.1
	750.9 [M−H]^−^	PE (p-16:0/20:4)	9.7 ± 6.5	7.5 ± 4.8	8.3 ± 8.0
	762.5 [M−H]^−^	PE (18:0/22:6)	4.5 ± 4.8	7.6 ± 5.4	0.4 ± 1.1
	766.6 [M−H]^−^	PE (18:0/20:4)	8.9 ± 5.9	9.0 ± 4.8	3.3 ± 3.1
	774.8 [M−H]^−^	PE (p-16:0/22:6)	0.6 ± 1.0	0.3 ± 0.8	0.6 ± 1.9
	790.8 [M−H]^−^	PE (18:0/22:6)	1.2 ± 2.3	2.5 ± 2.8	0.1 ± 0.3
SM	745.6 [M+COOH]^−^	SM (d18:1/16:1)	7.5 ± 3.6	14.5 ± 4.6	2.8 ± 1.8
	687.5 [M−CH_3_]^−^	SM (d18:1/16:0)	124.2 ± 27.0	169.4 ± 26.3	84.9 ± 18.3
	737.6 [M+Cl]^−^				
	747.6 [M+COOH]^−^				
	773.7 [M+COOH]^−^	SM (d18:1/18:1)	1.8 ± 2.4	7.6 ± 3.4	0.1 ± 0.3
	775.6 [M+COOH]^−^	SM (d18:1/18:0)	15.2 ± 6.6	29.9 ± 6.1	4.5 ± 3.1
	819.9 [M+Cl]^−^	SM (d18:1/22:1)	3.8 ± 3.0	9.2 ± 3.0	0.7 ± 1.7
	829.8 [M+COOH]^−^				
	821.7 [M+Cl]^−^	SM (d18:1/22:0)	9.9 ± 5.0	15.1 ± 5.1	5.3 ± 4.4
	832.0 [M+COOH]^−^				
	845.7 [M+Cl]^−^	SM (d18:1/24:2)	13.3 ± 4.6	20.5 ± 4.3	7.4 ± 3.7
	855.9 [M+COOH]^−^				
	847.8 [M+Cl]^−^	SM (d18:1/24:1)	38.3 ± 9.5	55.5 ± 6.9	25.2 ± 5.7
	857.8 [M+COOH]^−^				
PI	833.6 [M−H]^−^	PI (16:0/18:2)	17.5 ± 5.6	16.4 ± 6.3	12.6 ± 4.0
	835.7 [M−H]^−^	PI (16:0/18:1)	17.2 ± 5.4	16.0 ± 6.4	13.0 ± 4.4
	857.6 [M−H]^−^	PI (16:0/20:4)	18.8 ± 7.1	19.2 ± 5.5	13.4 ± 3.4
	861.8 [M−H]^−^	PI (18:0/18:2)	43.0 ± 12.7	41.8 ± 16.9	26.0 ± 8.2
	885.6 [M−H]^−^	PI (18:0/20:4)	115.6 ± 33.1	138.1 ± 47.1	80.1 ± 20.5
	909.7 [M−H]^−^	PI (18:0/22:6)	10.9 ± 2.8	16.1 ± 8.0	8.3 ± 2.0

Besides normal PEs, four plasmalogens PE (p-14:0/20:4), PE (p-16:1/20:4), PE (p-16:0/20:4) and PE (p-16:0/22:6) were identified at 9.23 min also. Plasmalogen is a kind of critical PL playing role in human health through participating in neuronal development and immune response, and acting as endogenous antioxidants (Braverman and Moser, 2012; Paul et al., 2019). Plasmalogen generally consists of a fatty alcohol with a vinyl-ether bond at the *sn*-1 position, and a polyunsaturated fatty acid at the *sn*-2 position of the glycerol backbone. According to the head groups, plasmalogens are mainly classified as PC plasmalogens and PE plasmalogens. Zhu et al. reported that the retention time of PLs in the same class was primarily decided by the length of the fatty acid chain and should be shortened with the increasing chain length of PL (Zhu et al., 2012). For this reason, two adjacent TIC peaks at 9.76 and 10.18 min were both attributed to the SMs with larger fatty acid chain differences. SM, also named sphingophospholipid, is a unique and important member of the PL family, in which the glycerol is substituted with sphingosine and the C-1 alcohol group of sphingosine is esterified to phosphorylcholine. In MS spectra, SM (d18:1/22:1), SM (d18:1/22:0), SM (d18:1/24:2), SM and (d18:1/24:1) with longer fatty acid chain were eluted from the column at 9.76 min, while SM (d18:1/16:1), SM (d18:1/16:0), SM (d18:1/18:1) and SM (d18:1/18:0) with shorter fatty acid chain were eluted at 10.18 min. These results were consistent with the conclusion of a previous report (Zhu et al., 2012). At the end of HILIC separation, PI molecular species were detected at 14.52 min. The dominating peak at *m/z* 885.6 was identified as the deproton ion [M-H]^−^ of PI (18:0/20:4). To sum up, a total of 31 molecular species of PLs, including 7 PCs, 10 PEs, eight SMs, and six PIs, were characterized in human plasma.

The ESI-MS ionization efficiency of different covalently linked compounds depends on their dipole potentials (Han and Gross, 2005). Because the zwitterionic polar head group of a PL molecule contributes the dominant dipole of the whole molecule, the ionization efficiency of different molecular species of PL in the same class is identical within the same experimental conditions. So the calibration curves for four PL classes quantification were plotted using the peak areas in extracted ion chromatogram (XIC) *versus* the concentrations of the external standards PC (18:0/20:4), PE (18:0/18:0), SM (d18:1/24:0), and PI (18:0/20:4) respectively using the weighted linear regression model. The LLOQ (lower limit of quantification) was defined with the signal-to-noise ratio (S/N) > 10. The quantification ions, calibration curve equations, the squares of linear correlation coefficient (*R* ^2^), linearity ranges, and retention times were given in Supplementary Table S1. With the assistant of external standard method, the concentrations of all characterized molecular species of PC, PE, SM, and PI in human plasma were successfully quantified and were listed in Table 2. In general, most PLs showed the highest concentrations in the CE group and the lowest concentrations in the HC group.

### Lipidomic Study of Plasma PLs

**FIGURE 2:**
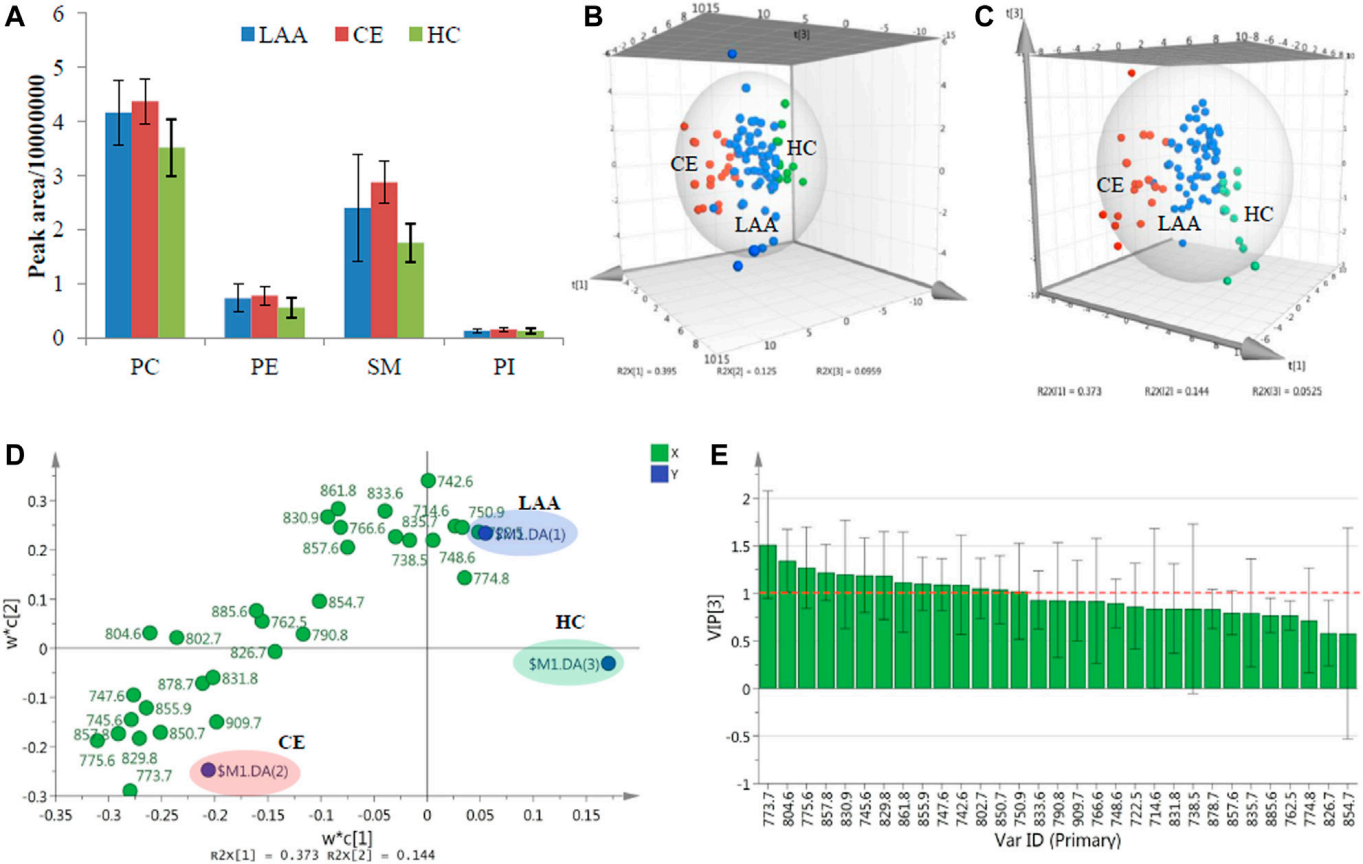
**(A)**. The mean TIC peak areas of four detected PL classes in LAA, CE, and HC groups; The 3D PCA score plot **(B)**, 3D PLS-DA score plot **(C)**, 2D PLS-DA loading plot **(D)** and VIP plot **(E)** of the determined concentration results of PL molecular species in LAA, CE, and HC groups.

In this work, 58 plasma samples from LAA patients, 19 plasma samples from CE patients, and 12 plasma samples from HC participants were treated and analyzed the described HILIC-MS methods. As shown in Figure 1A, the representative TIC chromatograms of LAA, CE, and HC subjects did not show visible differences. For comparison, the mean TIC peak areas of four different PL classes were calculated and shown in Figure 2A. One-way ANOVA method was employed to determine whether there is a significant difference. It was found that the peak areas of four PL classes, PC (red), PE (green), SM (yellow) and PI (blue) showed significant differences between any two groups (95% CI, *p*-value ≤ 0.05). In the LAA group, the peak areas of four PL classes were always higher than the HC group, but lower than the CE group. Referring to the calculated *p*-values, PC and SM showed more significant differences compared with PE and PI.

To further explore the detailed PL molecular species in charge of differences, all characterized plasma PL molecular species were quantified (Table 2). The final data were analyzed with a multivariate statistics method principal component analysis (PCA) for the lipidomic study. PCA is a dimensionality reduction method that can transform a large set of variables into several principal components containing most of the data matrix information. As shown in the three-dimensional (3D) PCA score plot in Figure 2B, which provided a visual representation of three components resulting from the concentration data of characterized PL molecular species from 89 plasma subjects, most of the subjects from the same colored group could cluster together, and the three classified groups could be separated from each other without the definition of group information in unsupervised analysis. Three outlier points from the LAA group lying outside the 95% confidence limit of Hotelling’s T^2^ value could be neglected. A supervised partial least squares-discriminant analysis (PLS-DA) was also performed, which is a descriptive and predictive modeling method based on PLS regression and has demonstrated great success in modeling high-dimensional datasets for disease classification in clinical practice (Lee et al., 2018). After dimensionality reduction, the concentration data of all detected plasma PL molecular species together with their group information could be demonstrated with three components in 3D PLS-DA score plot (Figure 2C). The data points from three colored groups clustered at the different areas in the graphic model. To preliminarily investigate the detailed PL molecular species bringing on the group classification, the loading plot of PLS-DA was shown in Figure 2D. The PL variables (X) situated in the vicinity of the dummy classes (Y) promise the highest discriminatory power among the LAA, CE, and HC groups. Besides the loading plot, the variable importance for the projection (VIP) plot in Figure 2E could summarize the importance of the variables for both description and discrimination. Generally, the VIP values larger than one indicate essential variables and those less than 0.5 indicate unimportant variables. In the end, a total of 14 crucial PL molecular species (4 PCs, two PEs, seven SMs, and one PIs) significantly contributing to the group differences could be provisionally obtained.

To further validate the differences of the obove 14 plasma PL metabolites among LAA, CE, and HC groups, one-way ANOVA was performed subsequently. After the statistical test, PC (18:0/18:2) (*m/z* 830.9), PI (18:0/18:2) (*m/z* 861.8), and PE (18:0/18:2) (*m/z* 742.6) was proved to show significant differences between the IS group (LAA and CE patients) and the HC group, but showed no difference between two IS subtype groups (LAA and CE). The only plasmalogen PE (p-16:0/20:4) (*m/z* 750.9) was proved to show no difference between any two groups. Except for these four PL molecules, the remaining ten PL metabolites, SM (d18:1/18:1) (*m/z* 773.7), PC (16:0/18:1) (*m/z* 804.6), SM (d18:1/18:0) (*m/z* 775.6), SM (d18:1/24:1) (*m/z* 857.8), SM (d18:1/16:1) (*m/z* 745.6), SM (d18:1/22:1) (*m/z* 829.8), SM (d18:1/24:2) (*m/z* 855.9), SM (d18:1/16:0) (*m/z* 747.6), PC (16:0/18:2) (*m/z* 802.7) and PC (16:0/22:6) (*m/z* 850.7), were proved to show significant differences between any two groups.

**FIGURE 3:**
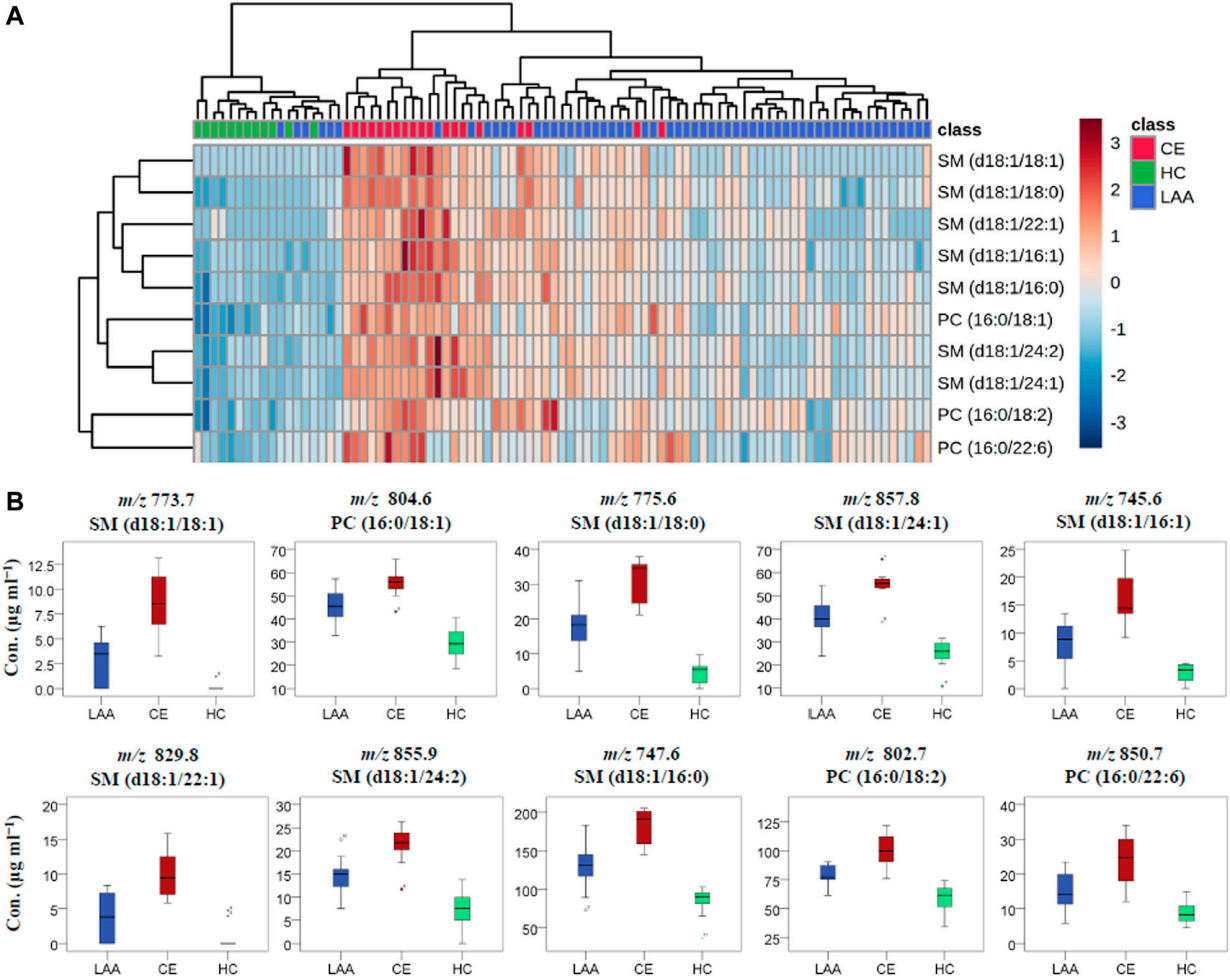
The visualized heatmap **(A)** and boxplots **(B)** of the determined concentration results of ten identified PL biomarkers in LAA, CE, and HC groups.

The visualized hierarchical clustering heatmap and boxplots of the concentration data of the ten potential PL biomarkers in LAA, CE, and HC groups were shown in Figures 3A,B, respectively. As we can see from these graphs, the PL concentrations were significantly different in each group, and all of these PL biomarkers showed the highest concentrations in the CE group and the lowest concentrations in the HC group. In terms of group clustering analysis with the heatmap in Figure 3A, most of the LAA and all CE subjects can be distinguished from HC subjects and clustered into a disease group according to the PL biomarker-based features of the 89 subjects. Further inspecting their detailed lipidomic features, most CE subjects can be differentiated from the LAA subjects. Among these validated PL biomarkers, there were 3 PC molecules and seven SM molecules. According to their fatty acid compositions, eight out of these ten biomarkers were found to be oleic acid-enriched PLs. In biomarker clustering analysis of the heatmap, these eight oleic acid-enriched PL biomarkers clustered together and showed different distribution characteristics at some point compared with two non-oleic acid-PL biomarkers PC (16:0/18:2) and PC (16:0/22:6). As a kind of most abundant ω-9 monounsaturated fatty acid (abbreviated as FA 18:1), oleic acid was reported to be correlated with greater risks of cardiovascular disease events and all-cause mortality in a large multiethnic cohort, which had relationships with IS to a certain extent (Steffen et al., 2018). In a recent study about the changes of serum metabolites in acute IS and its subtypes using MS-based metabolomics, oleic acid was found to be the most significantly changed metabolites between the IS group and the HC group according to the VIP values in orthogonal partial least squares-discriminant analysis (OPLS-DA) (Wang et al., 2021). In the condition of cerebral ischemia, oleic acid could be released from PLs and accumulate in plasma by the activation of phospholipase within a few minutes (Dhillon et al., 1997). In spite of this substantial evidence about the accumulation of oleic acid after IS stroke, the underlying pathogenesis remains unknown (Pilitsis et al., 2003). As an endogenous ligand of peroxisome proliferator-activated receptor-gamma (PPAR-γ), the neuroprotective effect of oleic acid through anti-inflammation during IS was demonstrated by Song et al. in rodent models (Song et al., 2019). To the best of our knowledge, this is the first report about the description of oleic acid-enriched PLs as potential biomarkers for diagnosing LAA and CE stroke.

Meanwhile, it was found that almost all of the identified SM metabolites are oleic acid-enriched and were considered as potential biomarkers in lipidomic study. Jiang et al. found that the plasma SM level was a risk factor for the development of atherosclerosis and was independently and positively related to coronary artery disease (Jiang et al., 2000). As we all know, atherosclerosis and coronary artery disease are closely linked with LAA and CE, respectively. It has long been known that the retention and aggregation of lipoprotein on the subendothelial matrix are the central pathogenic processes in atherogenesis, which may result from the increase of SM concentration. That is because, as the primary source of atherogenic lipoprotein in plasma, the increased SM concentration in slowly cleared lipoproteins can strengthen their susceptibility to arterial wall sphingomyelinase and then increase the atherogenic risk via lipoprotein retention and aggregation (Jeong et al., 1998). So the plasma SMs might be potential biomarkers for atherosclerotic LAA. Interestingly, CE patients in the current study showed a higher level of SM concentration than LAA patients did. Future studies looking into the underlying mechanisms are warranted to explain this phenomenon. At the same time, acid sphingomyelinase, which can protect against ischemic injury and ameliorate neurological deficits, was implicated in the happening of IS and proved to be a potential target in IS therapies (Mohamud Yusuf et al., 2019).

Additionally, many previous papers had reported up-/down-regulated levels of lipid metabolites in patients with acute IS (Liu et al., 2017; Sun et al., 2017; Yang et al., 2017). Seo et al. compared the level of metabolomic biomarkers between patients with the diagnosis of cardioembolic stroke and non-cardioembolic stroke and found a decreased level of LysoPC(16:0), LysoPC(18:0), and PC(38:7) (Seo et al., 2018). Though the direction of changes may differ across researches, the evidence showed the potency to discriminate different stroke subtypes using panels of metabolite biomarkers.

### Diagnosis With PL Biomarkers

Once the PL biomarkers were identified, their diagnostic abilities were subsequently evaluated. The differences between LAA or CE group with HC group were significant, and all of the identified PL biomarkers always showed significantly higher concentration in the IS group, including LAA and CE patients, compared with the HC group. In this study, the receiver operating characteristic (ROC) curve was employed to illustrate the diagnostic ability of a classification model with the PL biomarkers at all classification thresholds. The ROC curves for the IS stroke diagnosis with independent PL biomarker were plotted with the sensitivity against 1−specificity and given in Supplementary Figure S2. The area under the curve (AUC) in the ROC curve was calculated to measure the diagnostic ability. When AUC = 1, the biomarker can perfectly diagnose IS patients. When 0.5 < AUC <1, there is a high chance that the biomarker will diagnose IS stroke. In general, an accurate diagnosis could be achieved when the AUC value is higher than 0.900. Among these ten PL biomarkers, the AUC values were found to be in the range of 0.740–0.959 and determined to be higher than 0.908 for PC (16:0/18:1), SM (d18:1/18:0), SM (d18:1/24:1), SM (d18:1/16:1), SM (d18:1/16:0), and PC (16:0/18:2), which promise a lipidomic blood/plasma screening technique for early IS diagnosis.

**FIGURE 4:**
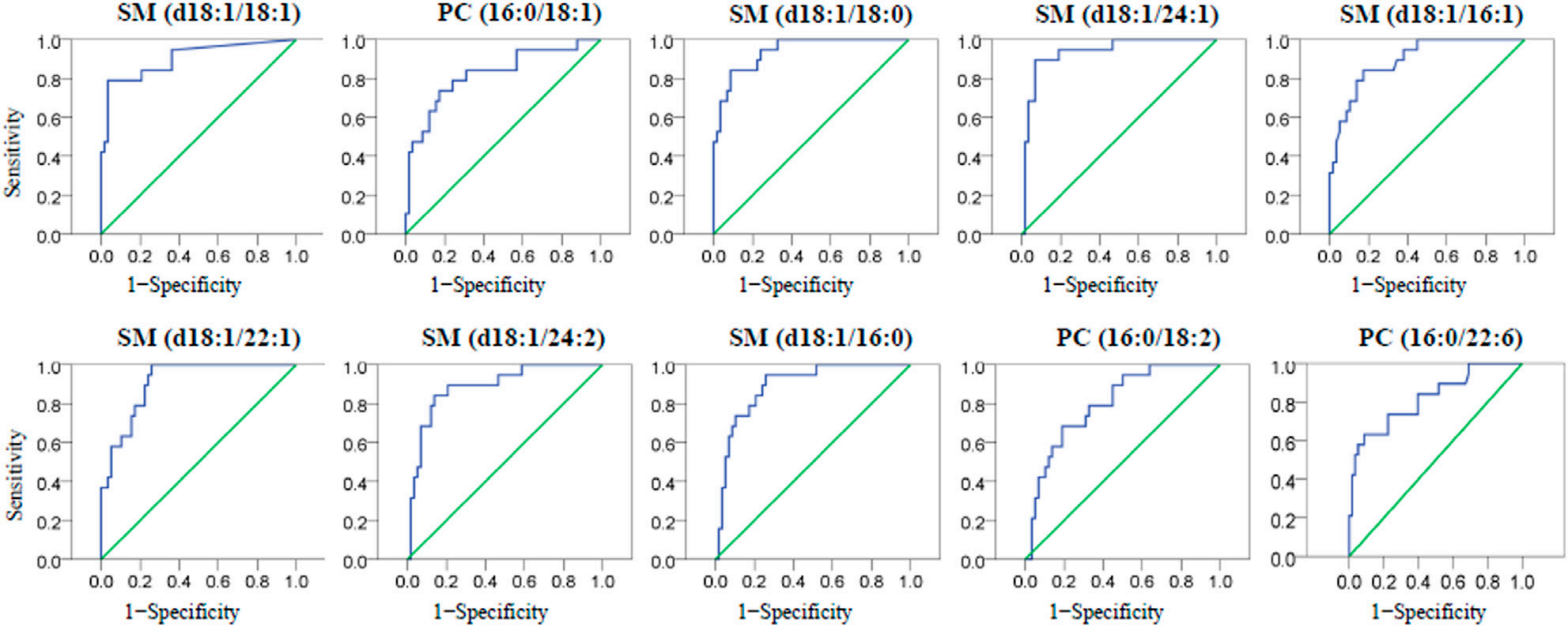
The ROC curves of then one-way ANOVA validated PL biomarkers for the diagnosis of CE from LAA participants.

Under the conditions of cerebral ischemia, the classification of LAA and CE subtypes becomes very important because they need different medication strategies to prevent patients from recurrence of ischemic events. To diagnose CE patients from LAA patients with the identified PL biomarkers, the ROC curves of the ten PL biomarkers were plotted and given in Figure 4, and the AUC values were calculated to be between 0.800 and 0.939. Finally, SM (d18:1/18:1), SM (d18:1/18:0), SM (d18:1/24:1) and SM (d18:1/22:1) with AUC value at 0.903, 0.939, 0.936 and 0.909 respectively, are probable to be applied as qualified criteria in the diagnosis of CE from LAA patients. For each diagnosis, the optimal cutoff value was determined with the largest value of Youden’s index = sensitivity + specificity −1, which meant the diagnosis had the highest sensitivity and specificity at this threshold. The cutoff plasma concentrations of SM (d18:1/18:1), SM (d18:1/18:0), SM (d18:1/24:1) and SM (d18:1/22:1) were determined as 6.375, 23.480, 49.815 and 5.785 μg ml^−1^ respectively for the distinguishment of CE patients from LAA patients. This work introduced a simple lipidomic-based blood test to provide complementary information to make a rapid and accurate diagnosis of acute IS subtypes, which is valuable and of potential significance to choose proper secondary prevention strategy to reduce the recurrence of IS.

### Suggestions of Future Studies

Besides LAA and CE, other TOAST subtypes are of equal significance and corresponding researches are guaranteed to obtain a comprehensive understanding. Furthermore, increasing evidence has shown that a substantial amount of strokes of undetermined etiologies are attributed to embolism from undetermined sources. It would be interesting to assess this hypothesis from the view of metabolism by evaluating the profile of phospholipids in plasma from patients with cryptogenic stroke. Another piece of suggestion is that, other metabolites, like amino acids, should also be assessed on their prominence as potential biomarkers in the diagnosis and differential diagnosis of stroke.

## Conclusion

Stroke is the second-leading cause of death, and the annual number of stroke-induced deaths and disabilities has substantially increased in the recent 3 decades (Feigin et al., 2021). In this study, molecular species of PL-targeted lipidomics together with the diagnosis of IS and its most common subtypes or etiologies: LAA and CE, were successfully conducted for the first time. Plasma PLs were selectively extracted and purified with a laboratory synthesized TiO_2_/KCC-1 material in SPE and subsequently determined in HILIC-MS analysis. In lipidomics, a total of 31 plasma PL molecular species were accurately characterized and quantified using an external standard method. With the help of statistical multivariate analysis, ten potential PL biomarkers SM (d18:1/18:1), PC (16:0/18:1), SM (d18:1/18:0), SM (d18:1/24:1), SM (d18:1/16:1), SM (d18:1/22:1), SM (d18:1/24:2), SM (d18:1/16:0), PC (16:0/18:2) and PC (16:0/22:6) were proved to be significantly different in plasma of LAA, CE and HC subjects. Some of these PL biomarkers showed promising capabilities for diagnosing IS and its subtype with proper sensitivity. Simultaneously, the plasma concentrations of oleic acid-enriched PLs were found to be positively related to IS and maybe a potential risk factor. Referring to the previously reported research, the hidden pathogenesis for IS might be due to the accumulation of the oleic acid-enriched SMs which can result in atherogenic lipoprotein aggregation in the human blood (Jeong et al., 1998). As a major source for PLs, these findings are helpful for dietary control and intervention. More lipidomic and pathological studies about IS and its etiologies still need to be conducted in the future to support and confirm these findings.

## Data Availability

The datasets presented in this study can be found in online repositories. The names of the repository/repositories and accession number(s) can be found in the article/Supplementary Material.
