# Supplementary material for: Screening of Phospholipids in Plasma of Large-Artery Atherosclerotic and Cardioembolic Stroke Patients With Hydrophilic Interaction Chromatography-Mass Spectrometry
Source: Front Mol Biosci. 2022 Jan 20;9:794057. doi: 10.3389/fmolb.2022.794057 (PMC8812958; doi:10.3389/fmolb.2022.794057)
Supplement: Supplementary file 1 [file DataSheet1.DOCX]

**Supporting Information**

Table S1. The calibration curves of PL standards in HILIC-MS analysis.

| PL | Quantificaiton ion (m/z) | Calibration curve equation | R^2^ | Linearity range  (μg mL^−1^) | RT  (min) |
| --- | --- | --- | --- | --- | --- |
| PC (18:0/20/4) | 778.6 [M+COOH]^−^ | y = 2E-05x - 6.2773 | 0.9934 | 0.5 - 250 | 8.8 |
| PE (18:0/18:0) | 746.6 [M-H]^−^ | y = 4E-05x - 8.2022 | 0.9910 | 0.5 - 250 | 9.3 |
| SM (d18:1/24:0) | 859.8 [M+COOH]^−^ | y = 4E-05x - 10.95 | 0.9912 | 2.5 - 250 | 9.7 |
| PI (18:0/20:4) | 786.6 [M-H]^−^ | y = 0.0008x + 3.5505 | 0.9973 | 2.5 - 250 | 14.5 |

RT: retention time

Fig. S1. Representative MS/MS spectra of some PL ion peaks


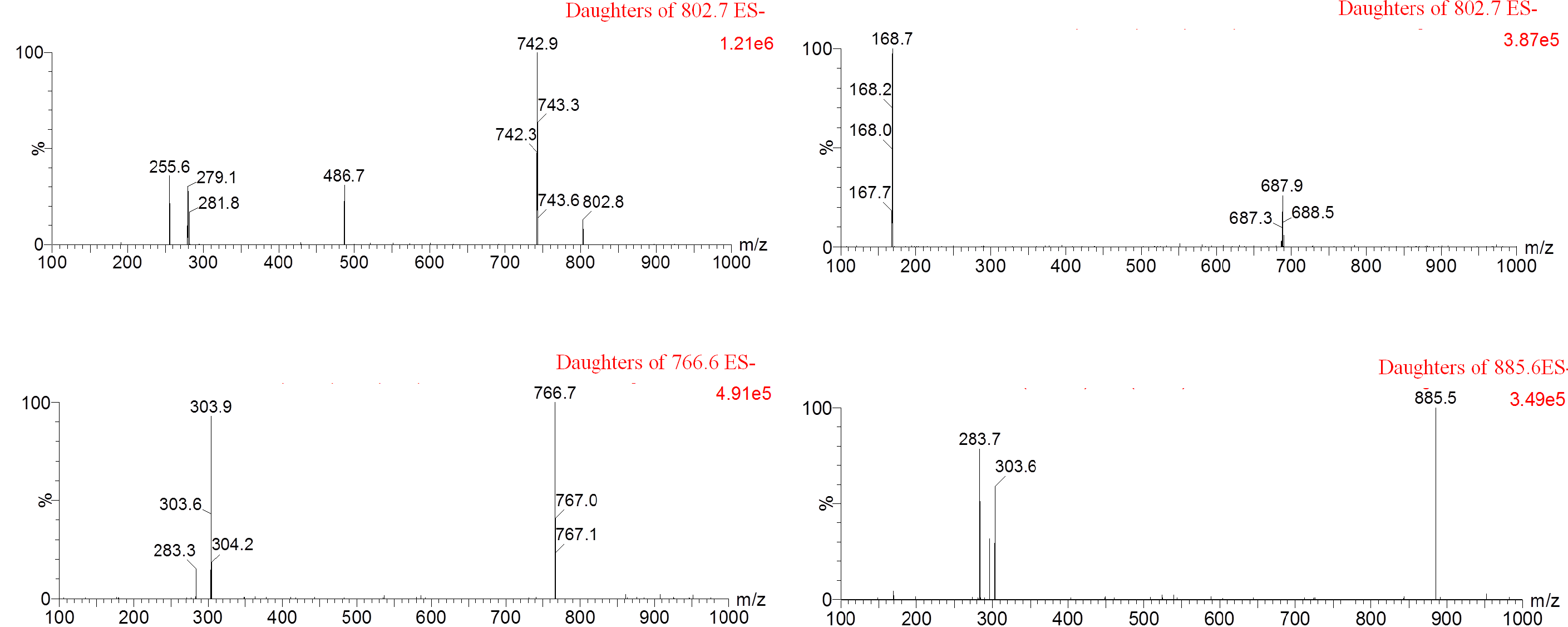


Fig. S2. The ROC curves of then one-way ANOVA validated PL biomarkers for the diagnosis of IS from HC participants.
